# Evaluation of Contamination in Milk Samples Pooled From Independently Collected Quarters Within a Laboratory Setting

**DOI:** 10.3389/fvets.2022.818778

**Published:** 2022-06-16

**Authors:** Chris J. Dean, Felipe Peña-Mosca, Tui Ray, Bradley J. Heins, Vinicius S. Machado, Pablo J. Pinedo, Luciano S. Caixeta, Noelle R. Noyes

**Affiliations:** ^1^Department of Veterinary Population Medicine, University of Minnesota, St. Paul, MN, United States; ^2^Department of Animal Science, University of Minnesota, St. Paul, MN, United States; ^3^Department of Veterinary Sciences, Texas Tech University, Lubbock, TX, United States; ^4^Department of Animal Sciences, Colorado State University, Fort Collins, CO, United States

**Keywords:** milk culture, composite samples, contamination, organic farms, dairy cows

## Abstract

The primary objective of this observational study was to evaluate the prevalence of contamination from independently collected quarter-level milk samples pooled in a laboratory and subjected to bacterial culture. To address this objective, weekly quarter-level milk samples were collected longitudinally from a cohort of 503 primiparous cows from five organic dairy farms during the first 5 weeks after calving. Individual quarter milk samples were pooled in a laboratory using aseptic technique (“lab-pooled”) and subjected to bacterial culture. In the sample set of 2,006 lab-pooled milk samples, 207 (10.3%) were classified as contaminated using a standard definition (i.e., growth of three or more distinct microorganisms). Subsequent culturing of corresponding quarter-level milk samples revealed that many of the contaminated lab-pooled sample results (i.e., 46.7%) were the result of intramammary infections with different pathogens across the quarters, rather than actual contamination within any single quarter (i.e., “true contamination”). The odds of true contamination were lower when the lab-pooled sample exhibited growth of three microorganisms compared to more than 3 microorganisms. Our findings suggest that pooling of quarter samples within a laboratory setting may yield lower rates of contamination compared to those previously reported from samples composited on-farm, but that current cut-offs to define contamination may need to be evaluated for use with lab-pooled samples. Further investigation of use of lab-pooled samples may be warranted to reduce costs while still providing useful scientific insight.

## Introduction

The collection and culturing of pooled milk samples is a common approach for identifying mastitis pathogens within lactating dairy cows, and has become common practice in both research and diagnostic studies related to udder health (1). However, some research questions may be well-suited to cow-level identification of bacteria in the udder, for example when investigating associations between systemic metabolic disturbances and mastitis or between host-associated microbiomes and mastitis. Pooled samples provide an affordable alternative to those collected and cultured from individual quarters (2), although the sensitivity of pooled samples has been estimated to be significantly lower than quarter-level samples (3). This is especially germane when screening for zero tolerance contagious pathogens such as *Staphylococcus aureus*, particularly in large herds when the prevalence is low and thus the negative predictive value would be high despite potential low sensitivity from pooled samples. Previous work has shown that subjecting pooled milk samples to matrix-assisted laser desorption ionization-time of flight mass spectrometry (MALDI-TOF MS) can provide additional benefit by allowing for the identification of different bacterial species, which more traditional on-farm culture does not typically provide (4). However, pooled samples have some limitations, including a dilution effect in which the mixing of milk from infected and non-infected quarters is thought to decrease the limit of detection of bacterial cells (1, 2). Despite this important limitation, pooled milk samples can provide a comprehensive taxonomic view of the microorganisms residing within milk (1, 5), allowing producers to tailor herd-level management strategies. To provide the most accurate and actionable culture results, special care must be taken to ensure samples are collected aseptically, so as to avoid the risk of contamination (6). In practice, this is difficult to do given the many opportunities for contamination that occur within a commercial dairy parlor during the collection of milk from all 4 quarters in a single vial. This practical consideration has prompted researchers and udder health specialists to recommend the collection and culturing of separate quarter-level samples (6).

**Figure 1:**
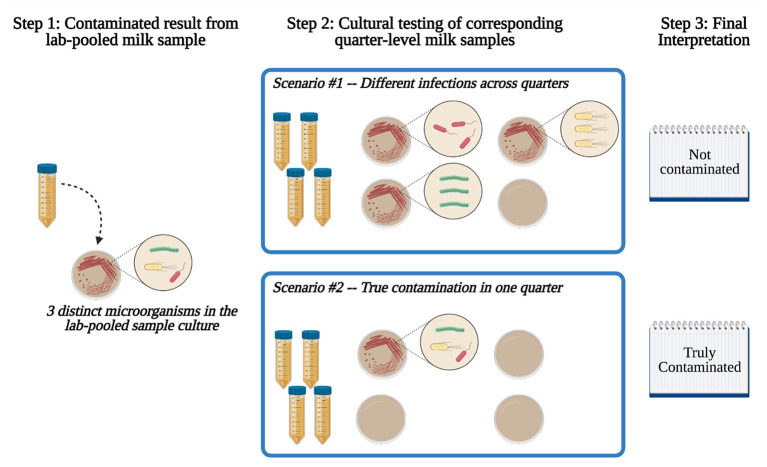
Possible scenarios and interpretations for a lab-pooled milk sample. In scenario #1, distinct pathogens are present across different quarter milk samples, resulting in a lab-pooled milk sample that should be classified as “not contaminated”. In scenario #2, three pathogens are present in a single quarter milk sample, resulting in a lab-pooled milk sample that should be classified as “truly contaminated”.

An alternative would be to aseptically collect quarter milk samples on the farm, and then pool them aseptically in a laboratory for pooled-level bacterial culture. Such an approach may strike an optimal balance between cost, accuracy, and information value for some research and production applications. However, this approach is not well-described in the literature and it is unknown how quarter-level collection and lab-based pooling may impact contamination rates. For example, many commercial and academic diagnostic laboratories define contamination as the growth of three or more distinct microbial species from a single sample (6). This threshold is used for both quarter and pooled milk samples, the latter of which typically refers to samples that are collected as a pool on-farm. Given that a set of four quarters can be differentially infected with distinct pathogens (7, 8), an alternative definition might be explored for pooled samples, particularly if contamination rates are low due to aseptic quarter-level on-farm sampling technique. Therefore, the primary objective of this analysis was to describe the prevalence of contamination in milk samples that were collected at the quarter level and then aseptically pooled prior to culture for mastitis pathogens (“lab-pooled” samples). We hypothesized that a large proportion of lab-pooled samples classified as contaminated would actually be the result of distinct bacteria present in the individual quarters that had been pooled together (Figure 1). We note that the analysis presented here was designed to address these objectives specifically for research purposes, and not for diagnosis of mastitis or herd-level management of udder health.

## Materials and Methods

### Study Design

The samples used in this study were collected as part of a larger observational study investigating associations between the cow udder microbiome and mastitis. The study was approved by the University of Minnesota Institutional Animal Care and Use Committee (Protocol Number: 1807: 36109A). In this longitudinal study, 503 primiparous Holstein cows were enrolled from 5 certified organic dairy farms in: Colorado (*n* = 162), Minnesota (*n* = 23; *n* = 66), New Mexico (*n* = 122), and Texas (*n* = 130) based on convenience sampling. Herd-level characteristics of each farm are presented in Table 1. Eligibility criteria for farm enrollment was based on willingness to participate, availability of electronic records, and organic certification. All nulliparous cows scheduled to calve within the study period were eligible for enrollment. Enrolled animals were sampled from March 2019 to January 2020, and we attempted to sample each enrolled animal weekly for up to 5 weeks postpartum. A total of 2,006 samples were collected across the 5 enrolled organic farms and the 503 enrolled nulliparous cows (Table 1). Most cows were sampled 4 (195/503) or 5 times (201/503), while 50, 16, and 36 were sampled once, twice or three times, respectively. There were also 5 animals sampled 6 or more times. The cows that were sampled fewer than 3 times typically were not sampled every week due to inability to locate them within the herd on the day of sampling.

**Table 1:** Herd characteristics for each farm enrolled in the study.

	**Farm A**	**Farm B**	**Farm C**	**Farm D**	**Farm E**
State	Colorado	New Mexico	Texas	Minnesota	Minnesota
Number of Cows Enrolled	162	122	130	23	66
Herd Size	1,200	3,000	1,500	100	275
Housing System^*^	Free stall barn	Dry lot	Dry lot	Free stall barn	Compost barn and out-wintering lot during winter
Enrollment Dates	July-Oct 2019	March-June 2019	Sept-Dec 2019	Aug-Dec 2019	March-May 2019

* *All enrolled farms provided access to pasture at least part of the year and cows consumed at least 30% of their dry matter intake from pasture when possible following organic farming regulations*.

### Sample Collection

Sample collection was performed by trained veterinarians and animal science students, and sample collectors were different between the enrolled farms. Quarter milk samples were collected from each animal on a weekly basis for 5 weeks after calving following guidelines described by the National Mastitis Council (6), unless the cows had severe clinical mastitis that prevented milk collection. All samples were collected prior to morning milking. Briefly, 3 to 4 streams of milk were discarded from each quarter and then each teat was scrubbed with a pre-moistened gauze square soaked in 70% ethanol solution (EtOH). Teats were then sampled in a clockwise direction, beginning with the left-rear quarter and ending with the right-rear quarter. When possible (i.e., when enough milk and/or colostrum was available and the cow was amenable to collection), approximately 10 mL of milk from each quarter was collected into separate 60 mL plastic vials. Samples collected in Minnesota were placed on ice until arrival at the University of Minnesota, where they were stored in a freezer at a temperature of −20°C. Samples collected outside of the state of Minnesota were placed on ice until frozen at a temperature of −20°C, and eventually shipped overnight on ice packs to the University of Minnesota.

### Milk Pooling

Prior to submission for bacterial culture, available quarter samples from each cow were pooled into a single sample using the following protocol. First, quarter samples were moved from −20°C to 4°C and allowed to thaw overnight. Then, thawed samples were placed inside a laminar hood that had been sterilized with 70% ethanol (EtOH) and subjected to 15 min of ultraviolet light exposure. Thawed milk samples were homogenized by shaking them back and forth. Next, 2 mL of milk was extracted from each quarter-level vial and dispensed into a single sterile plastic vial (“lab-pooled sample”). Quarter milk samples were then placed back inside −20°C. The lab-pooled samples were then submitted to the Udder Health Lab at the University of Minnesota (St. Paul, MN) for culture. Lab-pooled milk samples classified as contaminated were resubmitted at the quarter-level for culture, using the same overnight thawing and homogenization protocols as described above. A total of 207 lab-pooled samples were classified as contaminated, however 57 samples did not contain enough milk across at least 4 quarters, typically because the cow was difficult to sample on that day, or the quarters did not contain enough milk to obtain a full 10 mL. Of the remaining 165 samples, 150 had sufficient milk across all 4 quarters, resulting in a total of 600 quarter-level results. A total of five lab technicians and graduate students were involved in the pooling procedure, and each received hands-on training in aseptic technique by a PhD-level molecular biologist before performing pooling on the study samples.

### Bacterial Culture

For both pooled and quarter level samples, approximately 100 μL of milk was plated onto blood agar using a sterile cotton tip swab. Cultivation was performed under aerobic conditions by a single trained technician who regularly performs these procedures on submitted milk samples. Samples were incubated for 42–48 h at 37°C, as described previously (9, 10). A sample (lab-pooled or quarter-level) was considered positive for bacterial growth if it contained one or more colony forming units of any cultured isolate. Samples exhibiting growth of up to three distinct microorganisms were submitted for taxonomic identification using MALDI-TOF, while those exhibiting growth of more than three were not, based on the standard procedures of the lab performing the culture. Taxonomic assignments of cultured isolates were made using a MALDI-TOF mass spectrometer (MALDI Microflex LT Biotyper, Bruker Daltonics Inc.). Mass spectra profiles produced from each isolate were matched against the Biotyper reference library. Confidence scores were used to assign genus and species-level classifications, as described previously (10). Date of submission for bacterial culture was recorded and storage duration was calculated as the number of days that a given sample (i.e., lab-pooled or quarter level) was stored at −20°C, before processing.

### Definition of Contamination

Contamination of a lab-pooled milk sample was defined as growth of three or more distinct microorganisms (6). Contaminated lab-pooled milk samples were subjected to quarter milk culture and defined as truly contaminated if at least one quarter milk sample exhibited growth of three or more microorganisms. If none of the quarter milk samples contained growth of three or more organisms, the “contaminated” result from the pooled sample was considered to be the result of different infections across quarters (Figure 1).

### Sample Size Calculation

The samples used in this study were part of a larger research initiative to investigate potential associations between the udder microbiome and mastitis. Sample size estimations were calculated for the larger study and not the particular analyses presented in this paper. Therefore, *post-hoc* sample size calculations were performed to estimate the minimum sample size needed for an alpha (type I error) of 0.05; prevalence of true contamination of 46.7% (i.e., the percent of lab-pooled samples with 3 or more organisms in at least 1 quarter-level sample); and minimum difference of 20%. The resulting sample size was then multiplied by 1.2 to account for non-independence of observations within each farm, based on a previous estimate (10). This calculation yielded a minimum required sample size of 418 total lab-pooled samples, comprising 356 lab-pooled samples that contained more than 3 microorganisms (e.g., 85.3% of contaminated lab-pooled samples) and 61 lab-pooled samples that contained exactly 3 microorganisms (e.g., 14.7% of contaminated lab-pooled samples).

### Statistical Analysis

Statistical analysis and data cleaning were performed in R (https://www.r-project.org/; version 3.6.2). Summary statistics for animal and farm characteristics were generated to assess the integrity and accuracy of the data, and electronic and paper records were utilized to correct for any discrepancies identified (e.g., incorrect animal tags, farm names, dates of sample collection and calving dates). Two outcomes were modeled using the resulting data: 1) odds of contamination in the lab-pooled samples and 2) odds of true contamination in the lab-pooled samples. Both models were constructed using a mixed logistic regression modeling approach as implemented in the glmer function in the “lme4” package (11). For the odds of true contamination, the primary independent variable was the number of microorganisms detected in the contaminated lab-pooled milk samples (defined as more than three distinct microorganisms vs. three distinct microorganisms) and dependent variable was whether or not the sample contained “true contamination” (defined as presence of three or more organisms in at least one corresponding quarter sample). For the odds of contamination in the lab-pooled samples, the primary independent variable was storage duration. Both models were also offered storage duration and postpartum week as potential confounders. Confounding was assessed by comparing unadjusted and adjusted estimates for the primary independent variable in each model. Covariates that changed the estimates of the main exposure by more than 10% were maintained in each model. Cow and farm were forced into each model as random and fixed effects, respectively, in order to account for non-independence of observations. When Type III omnibus testing revealed a statistically significant association between the independent and dependent variable, pairwise comparisons were performed and Tukey adjustment was used to correct for multiple comparisons.

Additionally, Cohen's kappa statistic was also used to investigate the agreement beyond chance for results obtained from the lab-pooled samples (True = more than 3 microorganisms, False = 3) as compared to the results that would have been obtained from the corresponding quarter-level samples (True = 3 or more microorganisms in at least 1 quarter, False = <3 microorganisms in all quarter), as implemented in the “fmsb” package (12).

## Results

A total of 2,006 lab-pooled samples submitted for culture, 777 contained no growth, 776 contained 1 pathogen, 246 contained 2 pathogens, 43 contained 3 pathogens, and 164 contained more than 3 pathogens. For the quarter samples that were subjected to taxonomic identification (i.e., those with 3 or fewer distinct morphologic colonies in the relevant lab-pooled sample), the most common microorganisms included non-aureus Staphylococci, *Staphylococcus aureus* and *Streptococcus* spp. and *Streptococcus*-like organisms (Table 2), though the prevalence of each differed by farm.

**Table 2:** Prevalence (%) of microorganisms identified in 600 quarter milk samples submitted for culture.

**Microorganism**	**Herd A**	**Herd B**	**Herd C**	**Herd D**	**Herd E**
**NAS**	10/48 (20.8)	3/12 (25.0)	12/152 (7.9)	24/68 (35.3)	104/320 (32.5)
*Staph. chromogenes*	8/48 (16.7)	2/12 (16.7)	10/152 (6.6)	8/68 (11.8)	57/320 (17.8)
*Staph. sciuri*	0/48 (0.0)	0/12 (0.0)	0/152 (0.0)	0/68 (0.0)	16/320 (5.0)
*Staph. haemolyticus*	0/48 (0.0)	0/12 (0.0)	0/152 (0.0)	0/68 (0.0)	11/320 (3.4)
*Staph. hominis*	0/48 (0.0)	0/12 (0.0)	0/152 (0.0)	1/68 (1.5)	0/320 (0.0)
*Staph. xylosus/saprophyticus*	0/48 (0.0)	0/12 (0.0)	<1%	11/68 (16.2)	<1%
*Staph*. spp.	2/48 (4.2)	1/12 (8.3)	2/152 (1.3)	4/68 (5.9)	29/320 (9.1)
***Staph. aureus***	4/48 (8.3)	1/12 (8.3)	4/152 (2.6)	0/68 (0.0)	44/320 (13.8)
**SSLO**	2/48 (4.2)	3/12 (25.0)	15/182 (9.9)	16/68 (23.5)	43/320 (13.4)
*Strepto. dysgalactiae*	0/48 (0.0)	3/12 (25.0)	<1%	0/68 (0.0)	6/320 (1.9)
*Strepto. uberis*	0/48 (0.00)	0/12 (0.0)	0/152 (0.0)	0/68 (0.0)	8/320 (2.5)
*Aerococcus viridans*	0/48 (0.0)	0/12 (0.0)	0/152 (0.0)	9/68 (13.2)	23/320 (7.2)
*Aerococcus* spp.	0/48 (0.0)	0/12 (0.0)	0/152 (0.0)	7/68 (10.3)	<1%
*Enterococcus casseliflavus*	1/48 (2.1)	0/12 (0.0)	7/152 (4.6)	0/68 (0.0)	0/320 (0.0)
*Enterococcus mundtii*	1/48 (2.1)	0/12 (0.0)	4/152 (2.6)	0/68 (0.0)	<1%
*Enterococcus* spp.	1/48 (2.1)	0/12 (0.0)	4/152 (2.6)	0/68 (0.0)	0/320 (0.0)
**Gram-negative**	0/48 (0.0)	0/12 (0.0)	2/152 (1.3)	2/68 (2.9)	4/320 (1.3)
*Klebsiella spp*	0/48 (0.0)	0/12 (0.0)	0/152 (0.0)	1/68 (1.5)	0/320 (0.0)
*Pseudomonas* spp.	0/48 (0.0)	0/12 (0.0)	0/152 (2.9)	1/68 (1.5)	0/320 (0.0)
Gram-negative organisms	0/48 (0.0)	0/12 (0.0)	2/152 (1.3)	0/68 (0.0)	<1%
**Others**	2/48 (4.2)	1/12 (8.3)	29/152 (19.1)	0/68 (0.0)	61/320 (19.1)
*Bacillus* spp.	1/48 (2.1)	1/12 (8.3)	23/152 (15.1)	0/68 (0.0)	59/320 (18.4)
*Corynebacterium* spp.	1/48 (2.1)	0/12 (0.0)	7/152 (4.6)	0/68 (0.0)	<1%
Gram-positive rod	0/48 (0.0)	1/12 (8.3)	2/152 (1.3)	0/68 (0.0)	6/320 (1.9)
**No Growth**	26/48 (54.2)	2/12 (16.7)	78/152 (51.3)	29/68 (42.6)	36/320 (11.3)
**Contaminated**	4/48 (8.3)	3/12 (25.0)	20/152 (13.2)	11/68 (16.2)	92/320 (28.8)

*NAS, non-aureus Staphylococci; Staph, Staphylococcus; SSLO, Streptococcus spp. and Streptococcus-like organisms; Strep, Streptococcus*.

*Only microorganisms with a sample prevalence >1% are shown*.

**Figure 2:**
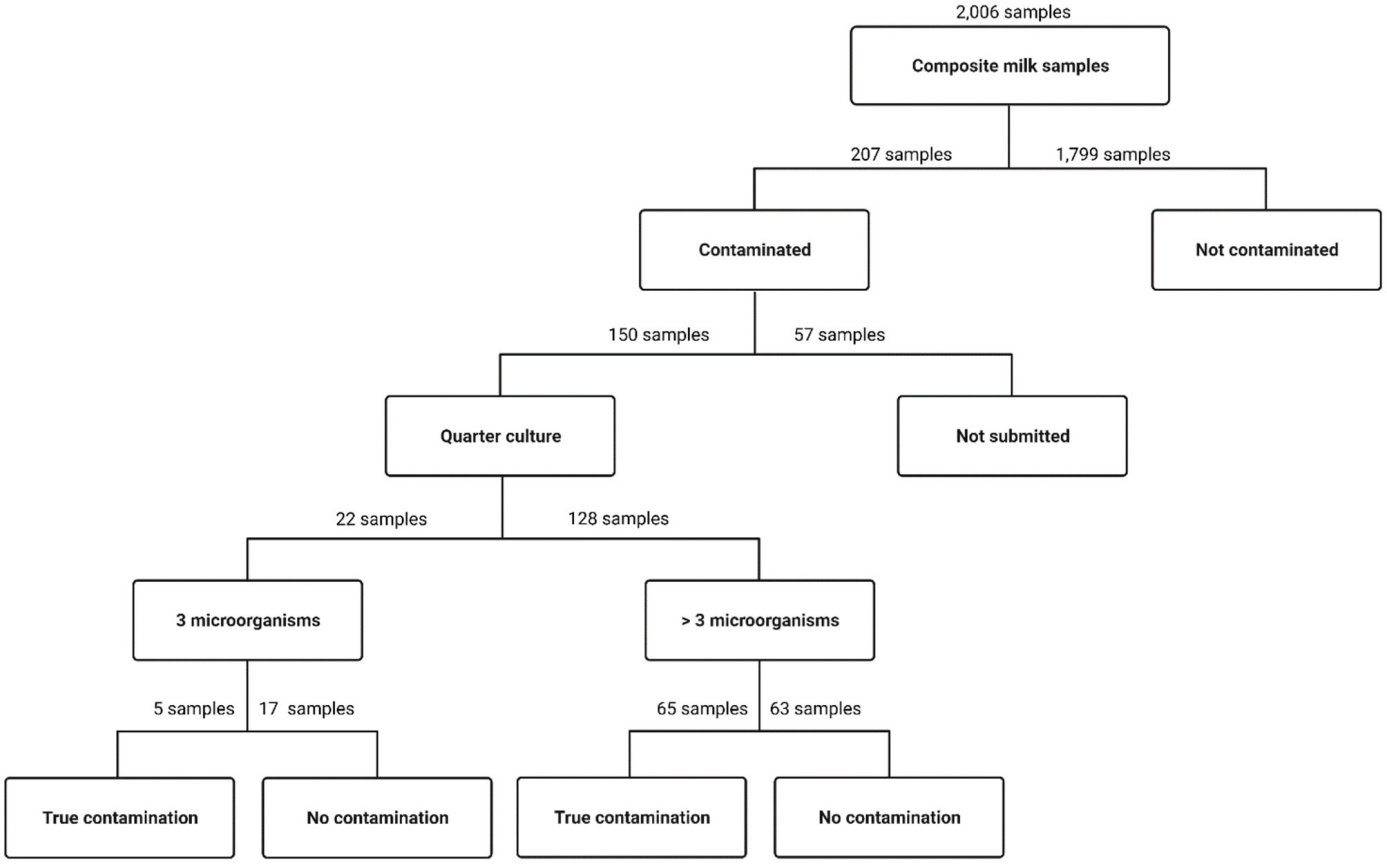
Flow chart describing the number of lab-pooled milk samples submitted for bacterial culture; the number of lab-pooled samples exhibiting growth of ≥3 microorganisms (“contaminated”) or <3 microorganisms (“not contaminated”); the number of lab-pooled samples submitted for follow-up quarter-level culture (“quarter culture”), divided into those that contained exactly “3 microorganisms” in the lab-pooled culture vs. those that contained “>3 microorganisms” in the lab-pooled culture; and the number that were classified as truly contaminated (“true contamination”, i.e., at least 1 quarter milk sample with ≥3 microorganisms) or not contaminated (“no contamination”, i.e., no quarters with ≥3 microorganisms).

Based on the standard definition of contamination (i.e., presence of three or more distinct organisms), 207 of the 2,006 cultured lab-pooled samples (10.3%) were classified as contaminated, with 43 of the 207 (20.8%) exhibiting growth of exactly three different microorganisms and 164 of the 207 (79.2%) more than 3 different microorganisms (Table 3). Of these 207 pooled milk samples, 150 contained enough residual milk in all 4 quarter-level samples to allow resubmission for quarter-level culture (Figure 2). The other 57 samples did not contain enough milk, typically because we could not obtain 10 mL due to cow temperament. Of these 150 lab-pooled samples, 70 (estimate = 46.7%, 95% CI = 38.7%−54.7%) were contaminated in at least one quarter sample (true contamination) and 80 (estimate = 53.3%, 95% CI = 45.3%−61.3%) were deemed to have no contaminated quarter samples (Table 3). The level of concordance for these results was estimated at a Kappa (95CI%) of 0.13 (−0.02–0.29) suggesting poor agreement.

**Table 3:** Number (%) of samples with contamination and true contamination status, stratified by the number of distinct organisms identified in the lab-pooled samples.

**Number of distinct organisms in lab-pooled sample**	**Number (%) of samples with the specified number of organisms**	**True Contamination^a^**	
		**Yes**	**No**
**Three or more**	207/2,006 (10.3%)	70/150 ^b^ (46.7%)	80/150 (53.3%)
Exactly three	43/207 (20.8%)	5/22 (22.7%)	17/22 (77.3%)
More than three	164/207 (79.2%)	65/128 (50.8%)	63/128 (49.2%)
**Two**	246 (12.3%)	N/A	12/80 (15.0%)
**One**	776 (38.7%)	N/A	11/80 (13.8%)
**Zero**	777 (38.7%)	N/A	9/80 (11.3%)

a *True contamination was defined as growth of 3 or more distinct microorganisms in at least one quarter milk sample that comprised the original contaminated pooled sample*.

b *Of the 207 lab-pooled samples classified as contaminated, 150 had enough residual milk to be cultured at the quarter level, and these comprise the denominator for the “True Contamination” values*.

Among the 80 lab-pooled samples with no contaminated quarters, 60.0% exhibited growth of 3 or more distinct microorganisms across all quarters comprising the lab-pooled sample, while 15.0, 13.8, and 11.3% exhibited growth of two, one or no microorganisms, respectively (Table 3). The proportion of lab-pooled and quarter-level samples classified as contaminated varied among farms (Table 4) and farm was a confounding variable across both multivariable models (Tables 5, 6). Postpartum week did not change the main estimates by >10% in any model, and therefore was not included in the final models. The odds of contamination in lab-pooled samples also differed significantly by farm (*P* < 0.05, Table 5). Pairwise comparisons between farms indicated that farm A had significantly lower contamination in pooled samples compared to other farms, while farm E had significant higher contamination than all other farms except D (*P* < 0.05, Table 5).

**Table 4:** Counts and percentages of lab-pooled samples with contamination, true contamination, and growth of at least one mastitis pathogen within each farm.

**Farm**	**No. of lab-pooled samples submitted for culture**	**No. (%) of lab-pooled samples that contained ≥3 distinct microorganisms**	**No. (%) of lab-pooled samples for which at least 1 associated quarter-level sample contained ≥3 distinct microorganisms (i.e., true contamination)^a^**	**No. (%) of non-contaminated lab-pooled samples with growth of any pathogen**
A	511	12/511 (2.3%)	3/12 (25.0%)	204/499 (40.9%)
B	568	25/568 (4.4%)	1/3 (33.3%)	328/543 (60.4%)
C	575	52/575 (9.0%)	14/38 (36.8%)	292/523 (55.8%)
D	89	20/89 (22.5%)	6/17 (35.3%)	47/69 (68.1%)
E	263	98/263 (37.3%)	46/80 (57.5%)	151/165 (91.5%)
Total	2,006	207/2,006 (10.3%)	70/150 (46.7%)	1,022/1,799 (56.8%)

a *Of the 207 lab-pooled samples classified as contaminated, 150 had enough residual milk to be cultured at the quarter level*.

**Table 5:** Odds of contamination^∧^ in lab-pooled samples based on mixed logistic regression modelling.

**Independent variable**	**Estimate (SE)**	**OR (95% CI)**	***P*-value**	**Type III**
Length of storage (months)	−0.28 (0.09)	0.76 (0.63–0.91)	0.003	
Farm†				<0.001
A^a^	*Reference*			
B^ab^	0.77 (0.40)	2.17 (0.99–4.72)	0.05	
C^bc^	1.44 (0.36)	4.21 (2.06–8.59)	<0.001	
D^cd^	2.39 (0.48)	10.86 (4.23–27.92)	<0.001	
E^d^	3.49 (0.39)	32.84 (15.41–70.00)	<0.001	

∧ *Contamination in lab-pooled samples was defined as growth of three or more distinct microorganisms*.

† *Different letters indicate significant differences between farms (P < 0.05)*.

*Random effects for cow (variance (SE)) = 1.006 (1.003), Intracluster correlation coefficient = 0.23*.

*A total of 2,006 lab-pooled results were included in the model*.

**Table 6:** Odds of true contamination ^∧^in the lab-pooled sample based on mixed logistic regression modelling.

**Independent variable**	**Estimate (SE)**	**OR (95% CI)**	***P*-value**	**Type III**
Length of storage (months)	0.40 (0.10)	1.48 (1.21–1.82)	<0.001	
Number of distinct microorganisms in lab-pooled sample				
More than three	*Reference*			
Three	−1.38 (0.59)	0.25 (0.08–0.80)	0.02	
Farm				0.11
A	*Reference*			
B	0.93 (1.42)	2.54 (0.16–41.31)	0.51	
C	1.14 (0.79)	3.14 (0.67–14.77)	0.15	
D	−0.96 (0.93)	0.38 (0.06–2.36)	0.30	
E	0.11 (0.81)	1.11 (0.23–5.40)	0.90	

∧ *True contamination in lab-pooled samples was defined as growth of three or more distinct microorganisms in at least one quarter-level sample Random effects for cow (variance (SE)) = 0 (0), Intracluster correlation coefficient = NA. A total of 150 lab-pooled results were included in the model*.

The number of microorganisms detected in the contaminated lab-pooled milk samples was associated with true contamination status (*P* = 0.02, Table 6). When three microorganisms were isolated from a lab-pooled milk samples, the odds of true contamination were lower than when more than three microorganisms were isolated from a lab-pooled sample (OR: 0.25, 95% CI: 0.08–0.80, Table 6).

The median length of storage was 279 days (range 68, 348) for quarter milk samples and 49 days (range 0, 268) for pooled milk samples. We observed a statistically significant association between storage duration and contamination in lab-pooled samples, with the odds of contamination decreasing with increased storage duration (OR (95% CI) = 0.76 (0.63–0.91), Table 5). Conversely, increased storage duration was associated with increased odds of true contamination (OR (95% CI) = 1.48 (1.21–1.82), Table 6).

## Discussion

A unique feature of this study was the aseptic pooling of milk from individually collected quarter milk samples. Using this approach, we reported an overall contamination prevalence of 10% (Figure 2 and Table 3), which is nearly three times lower than that reported from samples pooled on-farm (1). This may indicate that the aseptic pooling of quarter milk samples may not introduce additional contamination into the workflow.

Despite the relatively low prevalence of contamination in lab-pooled samples, we wanted to understand whether such results stemmed from true contamination or presence of distinct microorganisms across quarters. Therefore, we decided to perform culture on the quarter-level milk samples that corresponded to the contaminated lab-pooled samples. These quarter-level results revealed that only 46.7% of contaminated lab-pooled samples were actually contaminated in at least one quarter (Table 3). To explain this finding, we counted the total number of unique microorganisms cultured across each set of non-contaminated quarters (i.e., the set of quarters comprising each pooled sample). Based on this analysis, we found that a majority of the quarter-level sets exhibited growth of numerous distinct microorganisms across different quarters; when pooled together, these different quarter-level infections resulted in misclassification of the pooled sample as contaminated, when in fact the pooled sample contained different pathogens from each quarter (Table 3). The odds of a pooled sample being truly contaminated also differed depending on the definition used to define contamination in the lab-pooled sample (e.g., 3 vs. more than 3 microorganisms, Table 6), indicating that a definition traditionally used for quarter milk samples may not be appropriate for lab-pooled milk samples.

A major limitation of this comparison is that we likely failed to identify all contaminated milk samples in our sample set (Figure 2), resulting from the reduced sensitivity of bacterial culture on pooled samples (1). The analysis presented here was designed specifically to evaluate whether contaminated lab-pooled samples stemmed from true contamination at the quarter level vs. different bacteria across different quarters. Therefore, we did not evaluate non-contaminated lab-pooled samples and/or their respective quarter-level samples. Given this gap, future studies should include submission and analysis of quarter-level samples from non-contaminated pools (i.e., lab-pooled samples with <3 microorganisms), as well as a full description of the number, diversity and dis/concordance of microorganisms cultured from the lab-pooled and quarter-level samples. Additionally, we used a larger inoculum volume than is typically used for quarter-level milk culture (i.e., 100 vs. 10 μL), as a means of counteracting the potential dilution effect of pooled samples. Future studies should investigate the impact of inoculum volume on the sensitivity of culturing lab-pooled vs. quarter-level samples. Similarly, the impact of farm-level prevalence of different mastitis pathogens on the accuracy of lab-pooled sample results should be further evaluated, as farms with high prevalence of numerous pathogens may have a higher prevalence of different infections across quarters compared to farms with one dominant circulating pathogen. Such further analysis is also warranted given that our study reported results only for primiparous cows, which typically do not experience the same clinical mastitis dynamics as multiparous cows (7), particularly regarding pathogens such as *Staphlococcus* spp. (13), which were prevalent in our study population.

A major component of the lab-pooled workflow is the need to freeze the quarter-level milk until the lab-pooled results are finalized, which introduces differential storage time and an additional freeze-thaw cycle. Storage duration has been shown to impact milk culture results differentially across mastitis pathogens (2, 14)), although a head-to-head comparison of quarter-level vs. lab-pooled samples has not yet been conducted. Our findings included some milk samples that remained frozen for extended periods of time, far longer than is typically recommended by National Mastitis Council guidelines (6). However, this delay allowed us to examine the effects of storage duration on contamination in lab-pooled and quarter milk samples (2, 14). In lab-pooled milk samples, we observed an inverse relationship between storage duration and contamination; as storage time increased, the odds of contamination in lab-pooled samples decreased (Table 5). This finding may explain why 9% of the quarter-level samples did not yield any growth, despite the fact that their associated lab-pooled sample contained at least three distinct pathogens (Table 3). However, across all quarter milk samples, we observed the opposite relationship; as storage time increased, so did the odds of true contamination (Table 6). In other words, we lost microorganisms in pooled milk samples, but gained them in quarter milk samples. This observation is interesting, but not surprising as cell viability is thought to differ among various mastitis-causing pathogens when frozen for variable periods of time (2, 14). Thus, our results may be partially explained by differences in the number of freeze-thaw cycles and duration of storage between pooled and quarter samples. Furthermore, the bacteria implicated in contamination events are often different than those that cause intramammary infections within a given farm, and growth dynamics of different bacteria under frozen storage are known to differ (14). Finally, the magnitude of the dilution effect that occurs in pooled samples may change during extended frozen storage, due to a combination of evaporation and viability of various bacteria in the sample. Therefore, the combination of differential storage duration and bacterial taxa in the quarter vs. pooled samples may have initiated differential growth and detection opportunities between the two types of samples.

Future studies may wish to investigate the economic viability and practicality of the sample collection and screening procedures described in this study. Although the pooling of quarter samples in a laboratory may not be as efficient compared to collecting quarter milk into a single vial on-farm, it may represent an acceptable compromise between contamination and practicality. Furthermore, it could be hypothesized that pooling quarter level samples in the lab would result in significantly lower contamination rates than those pooled on-farm, with a much lower cost for bacterial culture. On a well-managed farm, many of the cultures would yield no-growth results, and if pooled, would equal the direct cost of a single culture; if not pooled, this would equal the direct cost of four cultures. In such cases, pooling could significantly reduce diagnostic costs, even if follow-up cultures were necessary for positive cows. However, it should be noted that direct diagnostic costs are only a small proportion of all costs associated with mastitis (15), and indeed a small reduction in diagnostic sensitivity (as can occur with dilution due to pooling) can greatly increase the overall cost of an effective mastitis testing program (16). Ultimately, the optimal approach to mastitis testing is highly dependent on farm-specific financial, management and biological factors; further evaluation is needed to identify the specific farm-level circumstances under which laboratory-pooled samples would be advantageous as part of a mastitis testing program.

The farms in this study represented a unique population of farms given their organic-certified status; additionally, they represented a range of herd sizes and management strategies (Table 1). Based on just these five farms, it is clear that the relative cost-benefit of lab-pooled samples may vary widely based on pathogen prevalence, as some of the farms had relatively high prevalence of some pathogens (Table 2). The interaction between pathogen prevalence, farm-level characteristics, and pooled vs. quarter-level sampling deserves closer attention, and future studies should consider including more farms with varying pathogen prevalence.

Milk collected at the quarter level on farm and then aseptically pooled in a laboratory could be a cost-effective and robust method to screen for zero-tolerance pathogens at the herd-level, as it obviates the limitations introduced by collecting quarter milk into a single vial; and allows for the retention of quarter-level data that might otherwise be lost or discarded using an on-farm pooling approach. However, further research is needed to understand the expected level of contamination in lab-pooled samples, particularly when collected and processed by different personnel. One of the strengths of this study was that samples were collected and processed by highly trained personnel, however this also means that the results may not be directly applicable to samples collected and processed under more typical conditions. In addition to applied uses, the lab-based milk pooling approach may have application within research studies that investigate epidemiological or biological questions focused on the cow level, and when funds are limited and information retention is critical.

## Conclusions

In this study, we described the prevalence of bacterial contamination in milk samples collected from individual quarters that were then pooled in a laboratory prior to culture. Our results, based on samples collected from first lactation Holstein heifers on five organic U.S. dairies, indicate that rates of contamination comparable to those of quarter milk samples are achievable when culturing composite milk samples with proper sampling and pooling techniques. Under these circumstances (i.e., proper on-farm sampling hygiene and lab-based aseptic pooling), pooled samples may provide useful information while reducing total cost. The short-term retention of quarter-level samples also provides an opportunity to retrospectively identify quarter-level intramammary infections if the corresponding pooled sample is found to exhibit bacterial growth. To maximize the utility of lab-pooled samples in large screening programs or research studies, further evaluation is needed to understand biases across pathogens, farms and sampling procedures; and to understand how different cutoffs for contamination in the lab-pooled sample impact diagnostic sensitivity and specificity and potential for misclassification.

## Data Availability Statement

The datasets presented in this study can be found in online repositories. The names of the repository/repositories and accession number(s) can be found below: https://github.com/TheNoyesLab/Comparing-lab-pooled-and-quarter-level-milk-culture

## Ethics Statement

The animal study was reviewed and approved by University of Minnesota Institutional Animal Care and Use Committee. Written informed consent was obtained from the owners for the participation of their animals in this study.

## Author Contributions

CD and FP-M wrote initial and final drafts of the manuscript, conducted statistical analysis, and collected milk samples. TR prepared milk samples for bacterial culture. CD, FP-M, NN, LC, BH, VM, and PP collected milk samples, enrolled farms and animals, and revised initial and final drafts of the manuscript. LC and NN obtained grant funding, designed the study, and revised initial and final drafts of the manuscript. All authors contributed to the article and approved the submitted version.

## Funding

This study was funded by the Organic Agriculture Research and Extension Initiative (OREI) from the National Institute of Food and Agriculture (Grant Number: 2018-51300-28563).

## Conflict of Interest

The authors declare that the research was conducted in the absence of any commercial or financial relationships that could be construed as a potential conflict of interest.

## Publisher's Note

All claims expressed in this article are solely those of the authors and do not necessarily represent those of their affiliated organizations, or those of the publisher, the editors and the reviewers. Any product that may be evaluated in this article, or claim that may be made by its manufacturer, is not guaranteed or endorsed by the publisher.
